# Still in Search for an EAAT Activator: GT949 Does Not Activate EAAT2, nor EAAT3 in Impedance and Radioligand Uptake Assays

**DOI:** 10.1021/acschemneuro.3c00731

**Published:** 2024-03-13

**Authors:** Lieve van Veggel, Tamara A.M. Mocking, Hubert J. Sijben, Rongfang Liu, Marina Gorostiola González, Majlen A. Dilweg, Jeroen Royakkers, Anna Li, Vijay Kumar, Yin Yao Dong, Alex Bullock, David B. Sauer, Hanne Diliën, Gerard J.P. van Westen, Rudy Schreiber, Laura H. Heitman, Tim Vanmierlo

**Affiliations:** † Department of Neuroscience, BIOMED Biomedical Research Institute, Faculty of Medicine and Life Sciences, Hasselt University, 3590 Hasselt, Belgium; ‡ Leiden Academic Centre for Drug Research (LACDR), Division of Drug Discovery and Safety, Leiden University, 2333 Leiden, The Netherlands; ⬡ Department of Psychiatry and Neuropsychology, Division of Translational Neuroscience, European Graduate School of Neuroscience, School for Mental Health and Neuroscience, Maastricht University, 6200 Maastricht, The Netherlands; ∥ Section of Psychopharmacology, Neuropsychology and Psychopharmacology, Faculty of Psychology and Neuroscience, Maastricht University, 6200 Maastricht, The Netherlands; ▼ University MS Center (UMSC), 3900 Hasselt-Pelt, Belgium; # Sensor Engineering Department, Faculty of Science and Engineering, Maastricht University, 6200 Maastricht, The Netherlands; ∇ Oncode Institute, Einsteinweg 55, 2333 Leiden, The Netherlands; ○ Centre for Medicines Discovery, Nuffield Department of Medicine, University of Oxford, OX3 7BN Oxford, U.K.; ◆ Nuffield Department of Clinical Neurosciences, Weatherall Institute of Molecular Medicine, University of Oxford, OX3 7BN Oxford, U.K.

**Keywords:** EAAT2, GT949, radioligand uptake, glutamate, transport, modulation

## Abstract

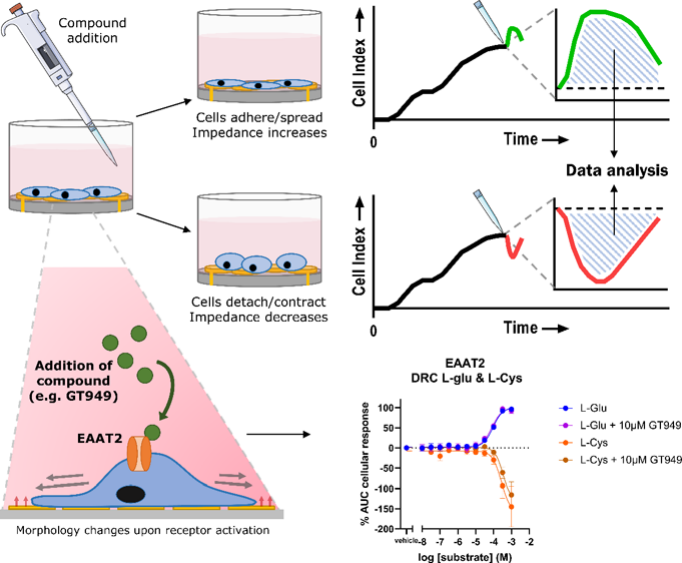

Excitatory amino acid transporters (EAATs) are important regulators of amino acid transport and in particular glutamate. Recently, more interest has arisen in these transporters in the context of neurodegenerative diseases. This calls for ways to modulate these targets to drive glutamate transport, EAAT2 and EAAT3 in particular. Several inhibitors (competitive and noncompetitive) exist to block glutamate transport; however, activators remain scarce. Recently, GT949 was proposed as a selective activator of EAAT2, as tested in a radioligand uptake assay. In the presented research, we aimed to validate the use of GT949 to activate EAAT2-driven glutamate transport by applying an innovative, impedance-based, whole-cell assay (xCELLigence). A broad range of GT949 concentrations in a variety of cellular environments were tested in this assay. As expected, no activation of EAAT3 could be detected. Yet, surprisingly, no biological activation of GT949 on EAAT2 could be observed in this assay either. To validate whether the impedance-based assay was not suited to pick up increased glutamate uptake or if the compound might not induce activation in this setup, we performed radioligand uptake assays. Two setups were utilized; a novel method compared to previously published research, and in a reproducible fashion copying the methods used in the existing literature. Nonetheless, activation of neither EAAT2 nor EAAT3 could be observed in these assays. Furthermore, no evidence of GT949 binding or stabilization of purified EAAT2 could be observed in a thermal shift assay. To conclude, based on experimental evidence in the present study GT949 requires specific assay conditions, which are difficult to reproduce, and the compound cannot simply be classified as an activator of EAAT2 based on the presented evidence. Hence, further research is required to develop the tools needed to identify new EAAT modulators and use their potential as a therapeutic target.

## Introduction

Excitatory amino acid transporters (EAATs) play a crucial role in the transport of several amino acids into the cell. Five subtypes of these EAATs exist; EAAT1–5.^1,2^ All EAATs are capable of transporting glutamate, which plays a crucial role in brain functioning due to its role as a neurotransmitter. EAAT2, where *SLC1A2* is the corresponding gene, is mainly expressed in astrocytes and to a lesser extent in other glial cells and neurons.^3^ In the central nervous system, its main role lies in combating glutamate excitotoxicity in extracellular space. EAAT3 (*SLC1A1*) is mainly expressed in neurons and oligodendrocytes. It is a unique subvariant in the sense that it can also transport cysteine in its deprotonated form.^4^

In general, EAATs act through an elevator-like mechanism to transport their amino acid of choice.^5^ Glutamate is transported against its concentration gradient by utilizing the gradients of sodium ions, potassium ions, and protons as a driving force for secondary active transport.^4,6^ EAATs are assembled as homotrimers, consisting of scaffold and transport domains.^7^ Various transmembrane helices form this complex structure, where the transport domains translocate the substrate binding site through the plasma membrane, whereas the scaffolding domains remain fairly rigid. Several structures have been solved in recent years describing both inward and outward facing states of EAAT2 and EAAT3.^5,8−10^

Despite their importance, the molecular mechanisms underlying the function and regulation of EAAT2 and EAAT3 are still not fully understood. Recent studies elucidated the role of these transporters in disease pathogenesis and EAATs might prove potential therapeutic targets in the context of neurodegeneration.^4,11−13^ Our goal is to contribute to the ongoing efforts to unravel the complex biology of EAAT2 and EAAT3, as well as to identify novel strategies, for treating neurodegenerative disorders.

In order to investigate the therapeutic potential of EAATs, a variety of strategies currently exists. For both EAAT2 and EAAT3, flox mice exist to create either overexpression or knockout by using the Cre-Lox system.^14,15^ Other genetic alterations exist in the form of lentiviral/adenoviral transduction or CRISPR/Cas9.^16,17^ Additionally, pharmacological interventions have been described. Several inhibitors with a variety of selectivities are available to investigate a loss of function of EAATs. Most ligands are based on the structure of the endogenous substrates of EAATs, such as TFB-TBOA. This compound is an aspartate analogue and acts as a competitive inhibitor, blocking the active site and thus transport of other amino acids. TFB-TBOA is fairly nonselective, acting on EAAT1–3.^18,19^ As opposed to inhibition, the activation of EAATs proves to be more difficult. However, the spider venom *Parawixia bistriata* was shown to stimulate glutamate uptake by Fontana et al.^20^ This information, together with structural information and virtual screening, GT949 has recently been described as a potential novel allosteric activator of EAAT2.^6^

Recently, Sijben and co-workers introduced a novel impedance-based assay that can detect l-glutamate uptake by EAATs in real time on whole cells.^21^ This assay relies on the fact that EAAT-mediated l-glutamate uptake leads to changes in cell morphology, which can be detected as changes in cellular impedance using the xCELLigence real-time cell analyzer. The observed changes are attributed to EAAT-mediated cell swelling as a consequence of increased intracellular levels Na^+^ and K^+^ ions upon l-glutamate uptake by EAATs.^21^ These cell-swelling effects could be blocked with the EAAT inhibitor TFB-TBOA, indicating that the observed changes in cell morphology in the impedance-based assay are EAAT specific. This high-throughput amenable technology is very sensitive and could even be used to assess the activity of EAAT1 mutants and their inhibition with allosteric inhibitor UCPH-101.^22^ Hitherto, allosteric enhancers have not been investigated with this technology.

For the present work, we aimed to investigate whether the reported effects of the allosteric modulator GT949 on EAAT2 and EAAT3 could be reproduced in this impedance-based assay. Ultimately, this assay could then be used for future drug discovery endeavors for allosteric enhancers on EAAT2 and EAAT3.

## Results and Discussion

Previous studies have shown the importance of EAATs as therapeutic targets and a recent study successfully identified novel compounds that activate EAAT2.^6^ In the present work, we show that these effects cannot be reproduced in the novel impedance-based whole-cell assay as well as radioligand uptake assays showing the lack of reproducibility regarding GT949.

### Impedance-Based xCELLigence Assay Shows No Activation of EAAT2 nor EAAT3 by GT949

**Figure 1:**
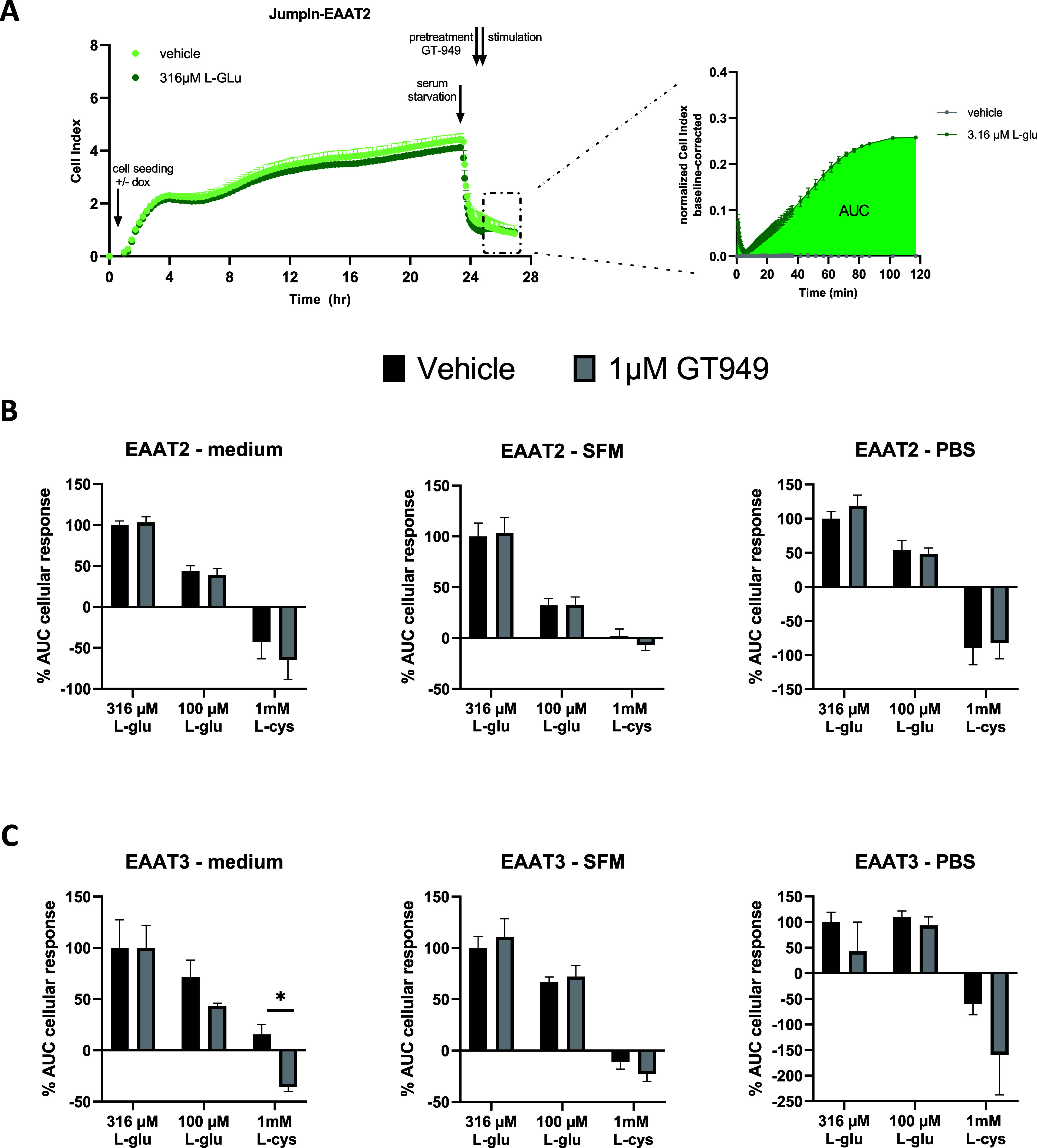
(A) Representative time trace of HEK293-JumpIn-EAAT2 cells following growth, starvation with PBS, pretreatment, and l-glutamate stimulation as measured by xCELLigence. Data were analyzed by taking the AUC over the first 2 h after stimulation. (B) EAAT2 or (C) EAAT3 mediated uptake of l-glutamate and l-cysteine after pretreated with vehicle or GT949 (1 μM) as measured by xCELLigence. Several starvation methods were used before the cells were pretreated with GT949 and stimulated, such as medium (left), serum free medium (middle), and PBS (right). Data are shown as the mean ± SEM of three individual experiments each performed in duplicate. *p < 0.05, one-way ANOVA with Dunnett’s posthoc test.

The recently described xCELLigence assay has been shown to be a reliable assay to study EAAT modulation and, therefore, is considered a reliable tool for testing the effects of GT949.^21^ Using the xCELLigence system uptake of l-glutamate by EAAT in HEK-JumpIn-EAATs will lead to changes in cell morphology, which are detected as an increase in cell index (CI) over the first 2 h (Figure 1A).

To exclude effects of any l-glutamate or l-cysteine already present in the medium prior to pretreatment and stimulation, three different starvation methods were applied. These included a replenishment with full medium, serum free medium (SFM), or PBS (Figure 1A). The representative time trace, shown in Figure 1A, displays an increase in CI upon stimulation with l-glutamate. In this setup, l-glutamate and l-cysteine transport by EAAT2 is not modulated upon GT949 (1 μM) stimulation following any of the starvation methods (Figure 1B). For completion, EAAT3 was also taken along since (Figure 1C), as stated in the literature, GT949 should not have an effect on this EAAT subtype.^6^ Indeed, apart from a minor effect on EAAT3 in the cysteine response when replenished with a full medium, the expected effect of EAAT2 activation was not observed.

**Figure 2:**
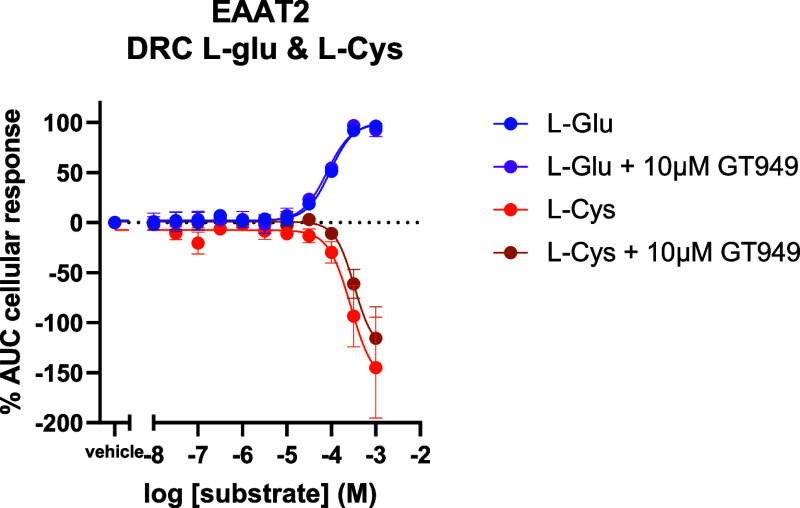
Dose response curve of the effects of GT949 pretreatment on the l-glutamate and l-cysteine response in EAAT2 expressing cells. Data are shown as the mean ± SEM of three individual experiments each performed in duplicate. One-way ANOVA with Dunnett’s posthoc test.

Second, the cellular response was tested in EAAT2 expressing cells after stimulation with a wide range of l-glutamate concentrations in the presence or absence of 10 μM GT949 (Figure 2). No significant changes in cellular response between the vehicle and 10 μM GT949 pretreated cells could be observed in the xCELLigence assay.

The xCELLigence data show no EAAT2, nor EAAT3 modulation upon treatment with GT949. Although inhibition of both these EAAT subtypes can be robustly shown in this assay, activation appears to not be as trivial.^21^ At this moment, there are few known allosteric modulators available, which could act as a positive control on EAAT2 and EAAT3 activation.^13^ Therefore, this does not allow us to conclude whether the assay cannot readily pick up activation or whether GT949 is failing in mechanistically activating EAAT2. Moreover, if the effect of GT949 can only be observed at low concentrations of l-glutamate, it could be overlooked in the impedance-based assay as it requires high micromolar concentration to detect EAAT2-mediated uptake. The findings in the xCELLigence assay require further research to reproduce the already described results; therefore, activation of EAAT2 by GT949 was assessed in radioligand uptake assays.

### Radioligand Uptake Assay Confirms That GT949 Does Not Modulate EAAT2 nor EAAT3 Activity

**Figure 3:**
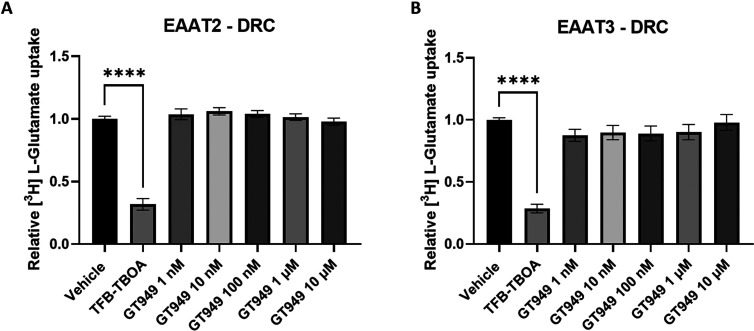
Radioligand uptake assay of EAAT2 (A) and EAAT3 (B). Dose response curves (DRCs) are shown. Dotted-line represent basal uptake by uninduced HEK-JumpIn cells. Data are shown as the mean ± SEM of two to four individual experiments each performed in at least duplicate. *p < 0.05, **p < 0.001, ***p < 0.005, ****p < 0.0001, one-way ANOVA with Tukey’s multiple comparison test.

**Figure 4:**
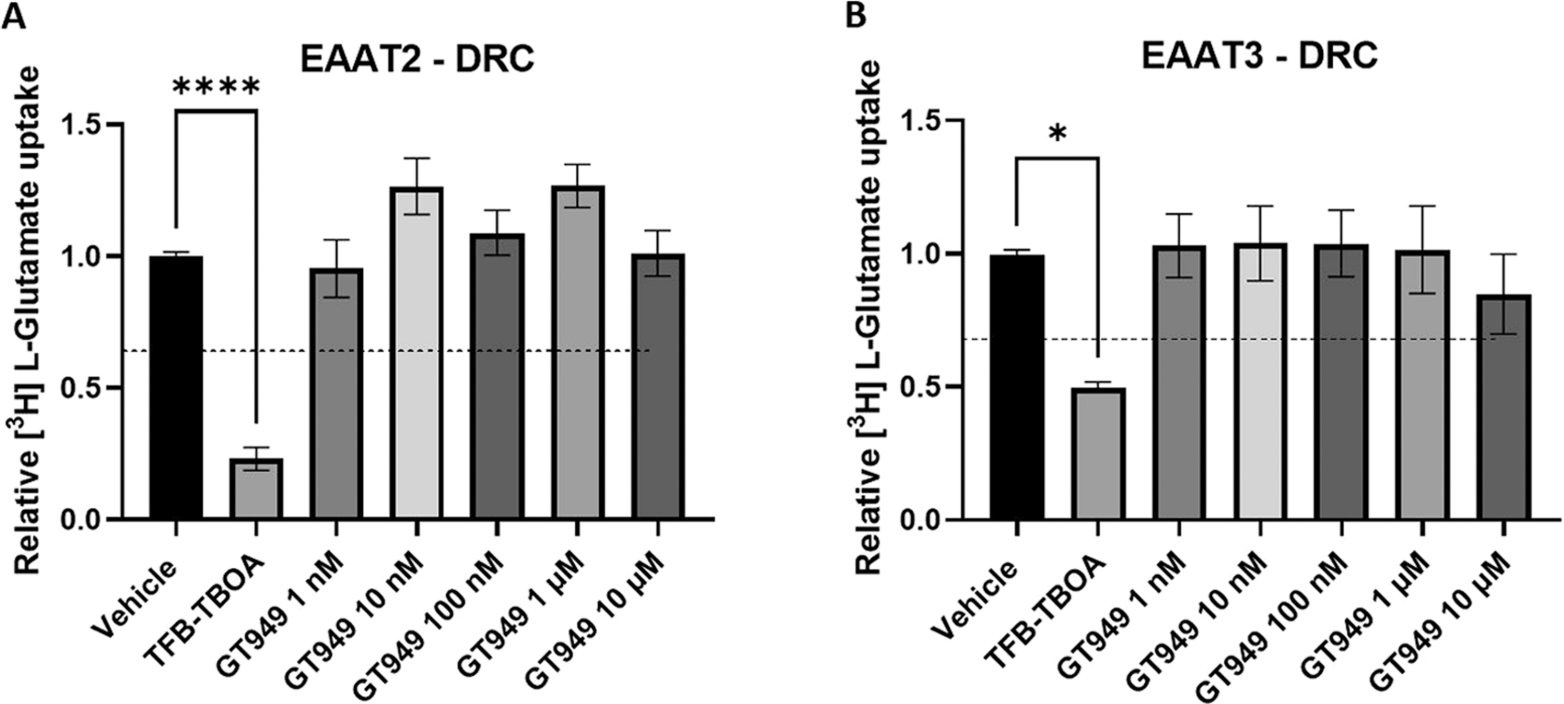
[^3^H]-l-glutamate uptake assay of HEK293-JumpIN-EAAT2 (A) and HEK-JumpIn-EAAT3 (B) cells showing the effect of GT949. Cells were induced with doxycycline to expressed the transporters. The dotted line represents the observed basal uptake of L-glutamate in uninduced cells (EAAT2: 0.65 ± 0.10, EAAT3: 0.68 ± 0.13). Data are shown as the mean ± SEM of three individual experiments each performed in at least duplicate. *p < 0.05, **p < 0.001, ***p < 0.005, ****p < 0.0001, one-way ANOVA with Tukey’s multiple comparison test.

To further validate whether GT949 truly activates EAAT2, radioligand uptake assays were performed. The protocols for the dose response curve (DRC) and kinetic assay (Figure 3) were adapted from previous work done in our lab.^23^ TFB-TBOA (10 μM) was taken as a negative control in all radioligand uptake assays. Indeed, TFB-TBOA, which has been described as a nonselective competitive inhibitor, acting on EAAT1, 2, and 3,^18,19^ clearly reduced radioligand (l-[3,4-^3^H]-glutamic acid) uptake in both EAAT2 and EAAT3 (Figure 3), in accordance with the normal signal-to-noise ratio found in the literature.^24,25^ The concentrations used for GT949 stimulation (1 nM–10 μM) all fall within the proposed DRC as described in the literature.^6^ Despite this, none of the GT949 concentrations used could modulate EAAT2 or EAAT3 compared with the control condition. In addition, our modeling results suggest stability of GT949 in the proposed EAAT2 allosteric binding pocket although lacking key interactions described previously (Supplementary Figure 1).^6^ This discrepancy further illustrates the lack of confidence in the proposed binding mode of GT949. However, as constructively suggested by the authors of the original description of the GT949 activity, Fontana et al.,^20^ this set up might not be fully optimal to identify activation as opposed to inhibition due to the cells being in suspension and a relatively long incubation time with the radioligand (1 h). To incorporate these changes, additional radioligand uptake assays (Figure 4) were performed to phenocopy the experimental conditions applied in and matching the description in the original description of GT949.^26^

In addition, uninduced cells were taken along as a negative control meaning that these cells were not treated with doxycycline, and therefore, expression of the transporter is not induced. The observed (basal) uptake in uninduced cells is due to EAATs endogenously expressed on HEK-JumpIn cells (*SLC1A1*: 6.8 TPM, *SLC1A2*: 0.3 TPM, *SLC1A3*: 34.6 TPM, *SLC1A4*: 11.3 TPM, *SLC1A5*: 289.8 TPM), as well as other transporters, involved in the glutamate system. Induction of EAAT2 in these cells significantly increased glutamate uptake, as expected, providing a sufficient assay window to detect any changes in uptake levels. TFB-TBOA was taken along as a pharmacological control, confirming that this assay setup can pick up changes in transporter activity through radioligand uptake. This new setup confirmed our previous findings where GT949 does not modulate EAAT2 nor EAAT3 at any of its concentrations (1 nM–10 μM) compared to the control. Furthermore, the purity of the compound has been tested and validated using LC/MS (Supplementary Figure 2). Together, these data show that the GT949 activation of EAAT2 is far from trivial. Assay setups have to be highly optimized and sensitive, and even then, it is difficult to pick up any effect of this compound on transport through radioligand uptake.

### GT949 Does Not Thermostabilize Purified EAAT2

**Figure 5:**
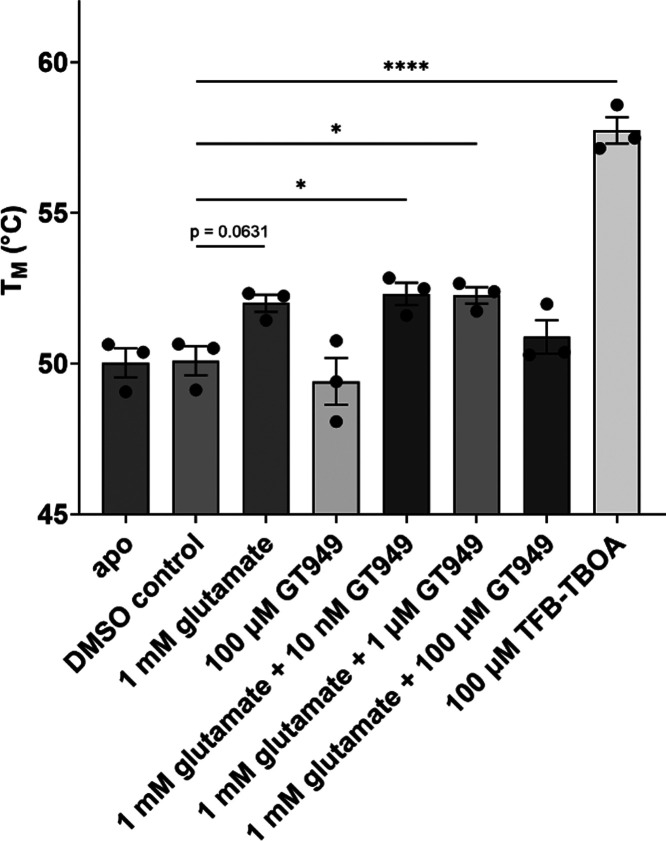
EAAT2 melting temperature with glutamate, GT949, and TFB-TBOA. Data are shown as mean ± SEM from three biological replicates. * *p* < 0.05, **** *p* < 0.0001, one-way ANOVA with Tukey’s multiple comparison test.

To probe the direct engagement of EAAT2 and remove confounding off-target effects by GT949, a nanoDSF thermal shift assay was performed with a purified transporter (Supplementary Figure 3). While EAAT2’s melting temperature significantly increased with TFB-TBOA (Δ*T*_M_ = 7.6 °C) and showed a trend to increase with glutamate (Δ*T*_M_ = 1.9 °C), the protein’s thermostability did not change in the presence of GT949 alone (Figure 5). In combination with glutamate, GT949 did not significantly change the transporter’s *T*_M_ relative to the glutamate-only condition. This suggests that the predominant effect was from glutamate rather than GT949. Collectively, the nanoDSF thermal shift assay confirmed that TFB-TBOA and glutamate bind EAAT2, but there was no clear evidence to support GT949’s interaction with the transporter.

## Conclusions

EAATs have been described as important transporters in different facets of amino acid transport, therefore posing a potential target in a wide array of neurodegenerative disorders. An interest has been taken in EAAT2 and EAAT3, especially due to their wide expression in glial cells and neurons. Unfortunately, compounds acting on these transporters, in particular, of the activating kind, are lacking. In order to perform relevant and robust drug discovery in academia, appropriate assays and confirmed tool compounds are required. Recently, an impedance-based whole-cell assay (xCELLigence) was described as a novel tool to investigate the uptake of EAATs. It has been shown to be sensitive to the inhibition of EAATs by a variety of compounds. However, activation remains to be investigated. The recently described allosteric activator GT949 is proposed as a useful candidate for this. GT949 has been described to activate EAAT2 in radioligand uptake assays in cell lines and primary astrocytes.

In the presented research, the effect of GT949 on HEK cells expressing either EAAT2 or EAAT3 was tested by using the impedance assay. Our data show that GT949 does not activate EAAT2 nor EAAT3 compared to the control. To further confirm whether this can be due to assay sensitivity, radioligand uptake assays were explored. Likewise, we were not able to show conclusive activation of EAAT2 or EAAT3 by GT949 based on radioligand uptake. Finally, results from the nanoDSF thermal shift assay do not support a direct interaction between GT949 and EAAT2. To conclude, these data provide a nuance of the already described effects of GT949 on EAAT2. We argue that specific assay conditions are required to pick up this activation, and therefore, it is not apparent to categorize GT949 as an EAAT2 activator. This urges the need for further development of modulators of EAAT activity in order to fully investigate and understand the therapeutic potential of these targets in neurodegenerative diseases. Furthermore, our work stresses the importance of reproducibility and assay robustness to truly study the effects of any compounds thought to act on EAATs, as well as other targets.

## Materials and Methods

### Chemicals and Reagents

The Jump in T-Rex HEK293 (JumpIn) overexpressing human EAAT2/3 were kindly provided by the RESOLUTE consortium (http://re-solute.eu).^21^l-glutamic acid monosodium salt monohydrate, l-cysteine, and doxycycline hyclate were purchased from Sigma-Aldrich (St. Louis, United States). (2*S*,3*S*)-3-[3-[4-(trifluoromethyl)benzoylamino]benzyloxy] aspartate (TFB-TBOA) was purchased from Axon Medchem (Groningen, The Netherlands). GT949 was purchased from Tocris Bioscience (Bristol, United Kingdom). xCELLigence PET E-plates 96 (ACEA Biosciences, San Diego, CA, United States) were purchased from BioSPX (Abcoude, The Netherlands). l-[3,4-^3^H]-glutamic acid (50.8 Ci/mmol) was purchased from PerkinElmer (Groningen, The Netherlands). All other chemicals were of analytical grade and were obtained from standard commercial sources.

### Cell Culture

JumpIn-EAAT cells were cultured as described previously.^21^ Briefly, cells were maintained in high glucose Dulbecco’s modified Eagle’s medium containing 10% (v/v) fetal calf serum, 2 mM Glutamax, 100 IU/ml penicillin, and 100 μg/mL streptomycin (culture medium) at 37 °C and 5% CO_2_. The cells were maintained in a culture medium supplemented with doxycycline to induce expression for 24 h prior to performing an experiment.

### xCELLigence Assays

The impedance-based xCELLigence assays were performed as recently described.^21^ In brief, JumpIn-EAAT cells were trypsinized, counted, and seeded (60,000 cells in 50 μL) in an E-plate already containing 1 μg/mL doxycycline to induce EAAT2/3 expression. The cells were left to rest for 30 min at room temperature and growth was monitored overnight (22 h). After these cells were either starved for 1 h in SFM or PBS or were refreshed with a normal medium and cell growth was monitored. This was followed by a 10 min pretreatment with GT949 (1 or 10 μM) as well as stimulation with the substrate (l-glutamate or l-cysteine) after which impedance was measured for at least 2 h.

### Radioligand Uptake Assay: 96-Well Assay

Cells were treated with doxycycline for 24 h after which they were trypsinized and counted. The cells were dissolved in the appropriate amount of culture medium to reach a concentration of 40,000 cells/25 μL.

For the DRC assays, Tris buffer (50 mM, 25 μL), compound (25 μL), and cells (25 μL) were incubated for 10 min at 37 °C. This was followed by the addition of radioligand (40 nM, 25 μL) and an incubation of 1 h at 37 °C. Incubation was terminated by rapid vacuum filtration through a 96-well GF/B filter plate using PerkinElmer Filtermate-harvester PerkinElmer (Groningen, The Netherlands). The filter plate was subsequently washed 10 times with ice-cold Tris buffer (50 mM). The plates were dried at 55 °C, after which MicroscintTM-20-cocktail was added (PerkinElmer, Groningen, The Netherlands). Intracellular radioactivity was determined by scintillation spectrometry using a MicroBeta^2^ 2450 Microplate Counter (PerkinElmer, Groningen, The Netherlands).^23^

### Radioligand Uptake Assay: 24-Well Assay

The dose–response assay was performed as described recently.^26^ Briefly, cells were seeded at 50,000 cells per well and incubated with doxycycline for 22 h. The cells were incubated with compound solution (Vehicle, GT949 or TFB-TBOA at various concentrations) for 10 min at RT. This was followed by the addition of radioligand and another incubation step of 10 min. Cells were washed, and lysis buffer was added for 20 min while being on a shaker. The lysate was transferred to a scintillation vial containing 3 mL of the scintillation fluid. Radioactivity was quantified using a Tri-Carb 2810 TR Scintillation Counter (PerkinElmer, Groningen, The Netherlands).

### SLC1A2 Expression and Purification

The full-length, codon-optimized gene of human SLC1A2 was cloned into pHTBV (kindly provided by Prof. Frederick Boyce, Harvard) with a C-terminal 3C PreScission site preceding EGFP, Twin-Strep, and 10-His tags. BacMam virus was generated using standard methods.^27^ Expi293F GnTI-cells in Freestyle 293 Expression Medium (Thermo Fisher Scientific) at 2 × 106 cells/ml, 37 °C, 8% CO_2_, and 75% humidity and transduced with the SLC1A2 P3 BacMam virus (3% v/v) in the presence of 5 mM sodium butyrate. The cells were incubated for a further 3 days at 30 °C. Cells were harvested by centrifugation at 1500*g* for 20 min, washed with phosphate-buffered saline, and flash frozen in liquid nitrogen before being stored at −80 °C.

SLC1A2-expressing cell pellets were resuspended in ice-cold extraction buffer (50 mM HEPES-NaOH pH 7.5, 200 mM NaCl and 5% glycerol) supplemented with cOmplete EDTA-free Protease Inhibitor (Roche). Cells were lysed with a Dounce tissue grinder followed by two passes through an EmulsiFlex homogenizer (Avestin), and solubilized in 1% w/v lauryl maltose neopentyl glycol (LMNG, Anatrace) with 0.1% cholesteryl hemisuccinate (CHS, Sigma) for 1 h at 4 °C. Lysate was clarified by centrifugation at 35,000*g* at 4 °C for 1 h. Protein was purified with Strep-Tactin XT 4Flow resin (IBA), tag removed with 3C PreScission protease overnight at 4 °C, and co-purifying proteins removed by reverse affinity purification with Strep-Tactin XT 4Flow or TALON resin (Takara Bio). The protein was further purified by size exclusion chromatography using a Superose 6 Increase 10/300 GL column (Cytiva) in gel filtration buffer (20 mM HEPES-NaOH pH 7.5, 150 mM NaCl, 0.0026% LMNG, and 0.00026% CHS).

### Thermal Shift Assay

Purified SLC1A2 in gel filtration buffer was incubated with 1 mM monosodium l-glutamate (Sigma) with 0 nM, 10 nM, 1 μM, or 100 μM GT949 (Tocris) or 100 μM TFB-TBOA (Tocris) for a minimum of 15 min at room temperature. The final protein concentration was 0.51 mg/mL. The sample was loaded into Prometheus standard capillaries (NanoTemper) and the intrinsic fluorescence at 350 and 330 nm was recorded by a Prometheus NT.48 (NanoTemper) as the samples were heated from 20 to 95 °C at 1 °C/min. The melting temperature (TM) was determined from the inflection point of the first derivative of 350 to 330 nm fluorescence ratio. Each biological replicate is a protein from an independent purification. Three to six technical replicates were performed per recording. TM of TFB-TBOA was used as an internal standard for protein quality.

### Data Analysis and Statistics

The experimental data obtained from the xCELLigence Assays were analyzed as described previously.^21^ In short, the CI values were obtained through the RTCA software and normalized to the time point prior to stimulation resulting in normalized CI values (nCI). Data were analyzed using GraphPad Prism (GraphPad Software, version 9.5.1., San Diego, CA, United States). Values were corrected for vehicle-induced, substrate-independent effects. After this, the nCI-vehicle-corrected responses were quantified by calculating the net area under the curve.

All other experimental data were also analyzed using GraphPad Prism 9.5.1. Relative ^3^[H]l-glutamate uptake was obtained by normalizing against the vehicle condition. After this, a one-way ANOVA was performed, including multiple comparisons to the vehicle condition. EAAT2 melting temperatures were compared by one-way ANOVA with Tukey’s multiple comparison test, with DMSO control against samples, as well as 1 mM glutamate against samples with both glutamate and GT949.

## Supporting Information Available

MD simulation of GT949 in the binding pocket of EAAT2, LC/MS analysis of GT949, and purification information regarding the thermal shift assay (PDF)
