# Supplementary material for: Still in Search for an EAAT Activator: GT949 Does Not Activate EAAT2, nor EAAT3 in Impedance and Radioligand Uptake Assays
Source: ACS Chem Neurosci. 2024 Mar 13;15(7):1424–31. doi: 10.1021/acschemneuro.3c00731 (PMC10995951; doi:10.1021/acschemneuro.3c00731)
Supplement: Supplementary file 1 — cn3c00731_si_001.pdf [file cn3c00731_si_001.pdf]

Supporting information

Still in search for an EAAT activator: GT949 does not activate EAAT2, nor EAAT3 in impedance and radioligand uptake assays.

Lieve van Veggel[1,3,5#], Tamara A.M. Mocking[2#], Hubert J. Sijben[2], Rongfang Liu[2], Marina Gorostiola González[2], Majlen A. Dilweg[2], Jeroen Royakkers[6], Anna Li[8], Vijay Kumar[8], Yin Yao Dong[9], Alex Bullock[8], David B. Sauer[8], Hanne Diliën[6], Gerard J.P. van Westen[2], Rudy Schreiber[4], Laura H. Heitman[2,7#], Tim Vanmierlo[1,3,5#]*.

[#]Equal contribution

[1]Department of Neuroscience, BIOMED Biomedical Research Institute, Faculty of Medicine and Life Sciences, Hasselt University, Hasselt, 3590, Belgium

[2]Leiden Academic Centre for Drug Research (LACDR), Division of Drug Discovery and Safety, Leiden University, Leiden, 1083 HV, The Netherlands

[3]Department of Psychiatry and Neuropsychology, Division of Translational Neuroscience, European Graduate School of Neuroscience, School for Mental Health and Neuroscience, Maastricht University, Maastricht, 6200 MD, The Netherlands

[4]Section of Psychopharmacology, Neuropsychology and Psychopharmacology, Faculty of Psychology and Neuroscience, Maastricht University, Maastricht, 6200 MD, The Netherlands

[5]University MS Center (UMSC), Hasselt-Pelt, 3900, Belgium

[6]Sensor Engineering Department, Faculty of Science and Engineering, Maastricht University, Maastricht, 6200 MD, The Netherlands

[7]Oncode Institute, Einsteinweg 55, Leiden, 2333 CC, The Netherlands

[8]Centre for Medicines Discovery, Nuffield Department of Medicine, University of Oxford, Oxford, OX3 7BN, United Kingdom

[9]Nuffield Department of Clinical Neurosciences, Weatherall Institute of Molecular Medicine, University of Oxford, Oxford, OX3 7BN, United Kingdom

*Corresponding author: tim.vanmierlo@uhasselt.be

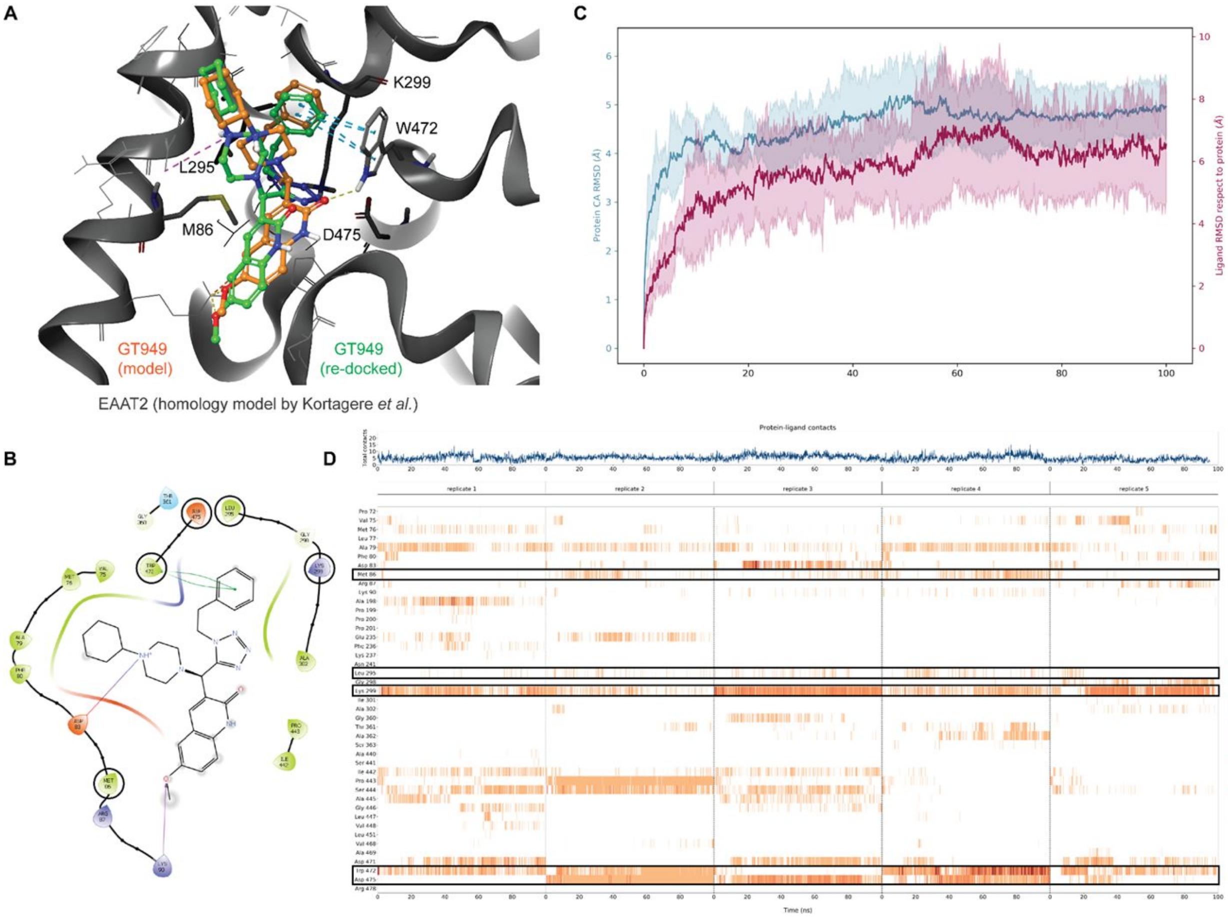

Supplementary Figure 1. GT949 stability assessment in the allosteric pocket defined by Kortagere et al. (a) GT949 redocking. The EAAT2 homology model generated by Kortagere et al. was prepared in Schrodinger Maestro v11.7 using protein preparation wizard with default settings. Similarly, GT9494 protonation and tautomerization was handled using LigPrep. GT949 was redocked on EAAT2 using Glide docking with Standard precision and the original pose (in orange) as centre of the docking box, where 5 poses were generated for each tautomer used as input. From the docking results, the pose with the highest docking score and most similar pose to the original one was selected. This re-docked pose (in green) had a Glide docking score of -4.08 and made the following interactions: pi-pi stacking (dashed blue line) with W472, salt bridge (dashed pink line) with D83, and hydrogen bond (dashed yellow line) with K90. The strongest of these interactions are also represented in a 2D diagram in (b). Compared to the original GT949 pose presented by Kortagere et al., the re-docked pose presented here lacks interactions with L295 and M86, which is a result of GT949 not accessing the allosteric pocket as deeply. (c) Molecular Dynamics (MD) simulations. To analyse the stability of the re-docked GT949 pose, five replicates od 100 ns MD simulations were performed in Desmond v6.6. For this, the complex system was embedded in a POPC bilayer membrane and hydrated with SPC waters. Moreover, charges were neutralized with Cl- ions and NaCl salt was added at physiological concentration of 0.15M. The system was minimized and equilibrated as per default Desmond settings, and five production replicates were run on OPLS4 force field and NPγT ensemble at 300 K and 1 bar with randomized initial velocities for 100 ns, with a recording interval of 100 ps. Root-mean square deviation (RMSD) was computed for the protein alpha-carbons (CA) and for the ligand with respect to the protein for each replicate, and the mean ± standard deviation was plotted in (c). Both the protein and the ligand showed relatively good stability over the 100 ns of the simulation. However, the increase in ligand RMSD suggests that the original pose is slowly replaced by a more stable one. (d) The protein-ligand interaction map suggests that in most of the replicates the more stable ligand conformation is stabilized by interactions with K299, W472, and D475 rather than M86 and D295, of which the former is at the entrance of the pocket and the latter deep inside it.

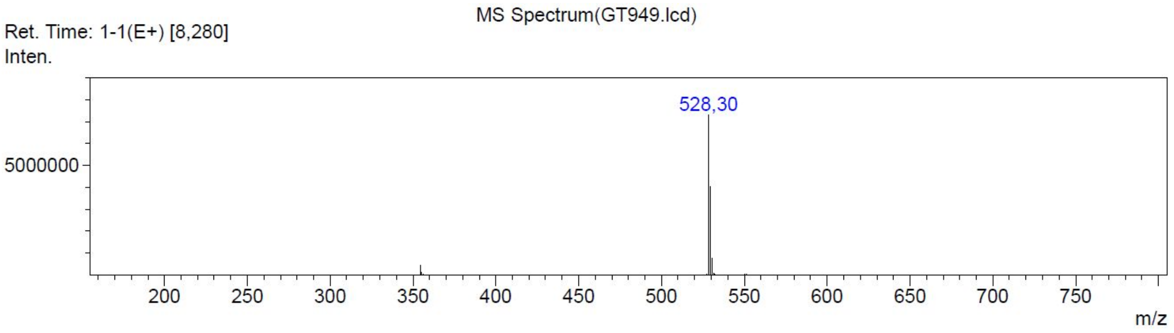

Supplementary Figure 2. LC/MS analysis of GT949. The spectrum shows a clear peak at m/z 528.30 (retention time of 8,280 min) with 99,6% purity indicating that the sample is pure.

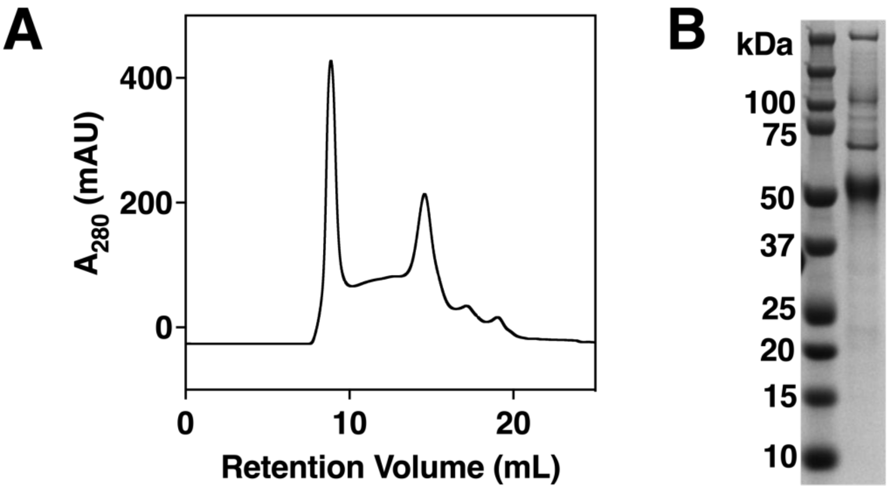

Supplementary Figure 3. Purification of EAAT2. (a) Preparative SEC of EAAT2 following Strep-Tactin XT purification. (b) Representative SDS-PAGE of purified EAAT2.
